# *Mycobacterium leprae* Infection in Ticks and Tick-Derived Cells

**DOI:** 10.3389/fmicb.2021.761420

**Published:** 2021-10-27

**Authors:** Natthida Tongluan, Layne T. Shelton, J. Hunter Collins, Patrick Ingraffia, Gregory McCormick, Maria Pena, Rahul Sharma, Ramanuj Lahiri, Linda B. Adams, Richard W. Truman, Kevin R. Macaluso

**Affiliations:** ^1^Department of Pathobiological Sciences, School of Veterinary Medicine, Louisiana State University, Baton Rouge, LA, United States; ^2^Department of Microbiology and Immunology, College of Medicine, University of South Alabama, Mobile, AL, United States; ^3^United States Department of Health and Human Services, Health Resources and Services Administration, National Hansen’s Disease Program, Baton Rouge, LA, United States

**Keywords:** *Mycobacterium leprae*, ticks, tick-derived cells, infection, transmission

## Abstract

Leprosy is a zoonosis in the southern United States involving humans and wild armadillos. The majority of patients presenting with zoonotic strains of *Mycobacterium leprae* note extensive outdoor activity but only rarely report any history of direct contact with wild armadillos. Whether *M. leprae* is transmitted to new vertebrate hosts through the environment independently or with the aid of other organisms, e.g., arthropod vectors, is a fundamental question in leprosy transmission. The objectives of this study were to assess the potential for ticks to transmit *M. leprae* and to test if viable *M. leprae* can be maintained in tick-derived cells. To evaluate tick transmission, nymphal *Amblyomma maculatum* ticks were injected with isolated *M. leprae.* Infection and transmission were assessed by qPCR. Ticks infected as nymphs harbored *M. leprae* through vertical transmission events (nymph to adult and adult to progeny); and, horizontal transmission of *M. leprae* to a vertebrate host was observed. *Mycobacterium leprae* DNA was detected in multiple tick life cycle stages. Likewise, freshly isolated *M. leprae* (Thai-53) was used to infect a tick-derived cell line, and enumeration and bacterial viability were assessed at individual time points for up to 49 days. Evaluations of the viability of long-term cultured *M. leprae* (Thai-53 and Br4923) were also assessed in a mouse model. Tick-derived cells were able to maintain viable *M. leprae* over the 49-day course of infection and *M. leprae* remained infectious within tick cells for at least 300 days. The results of this study suggest that ticks themselves might serve as a vector for the transmission of *M. leprae* and that tick cells are suitable for maintenance of viable *M. leprae* for an extended period of time.

## Introduction

Hansen’s Disease, more commonly known as leprosy, is caused by an obligate intracellular bacillus, *Mycobacterium leprae*. Presenting as a granulomatous infection that involves the skin and peripheral nerves, leprosy can deform and disable individuals if not properly and promptly treated (Walker and Lockwood, 2006). Current disease control strategies depend on the early diagnosis and treatment of new leprosy cases. However, poor diagnostic criteria for leprosy and the stigma that surrounds it act as barriers to both diagnosis and treatment (Predaswat, 1992; World Health Organization [WHO], 2019). A lack of understanding of how leprosy is spread also confounds efforts to eliminate the disease, as transmission is thought to occur mainly through exposure of susceptible individuals to bacilli shed from untreated or asymptomatic cases, though as yet the exact mechanisms are still unclear (Davey, 1974; Scollard, 2005). Environmental contamination and zoonotic transmission from non-human reservoirs also have been implicated (Blake et al., 1987; Desikan and Sreevatsa, 1995; Truman and Fine, 2010; Truman et al., 2011; Sharma et al., 2015; Avanzi et al., 2016; Mohanty et al., 2016; Tio-Coma et al., 2019).

Though the majority of new cases of leprosy in the United States can be associated with international travel to regions where the disease is more prevalent, 38% of the new cases identified in 2014 were considered autochthonous (HHS, 2015) and many of those appear to have evolved through zoonotic transmission of leprosy bacilli. Nine-banded armadillos (*Dasypus novemcinctus*) in the southern United States, as well as in Central and South America, harbor a natural infection with *M. leprae* that was likely introduced to the animals sometime after colonization of the *New World.* Large numbers of infected armadillos were known to exist in the southern United States well before the animals were ever used in leprosy research, and recent studies confirm that armadillos are a source of infection for many leprosy cases in the United States (Truman et al., 1986; Sharma et al., 2015). While direct contact with the blood or tissues of infected armadillos is recognized as an important risk factor for zoonotic transmission of *M. leprae* from armadillos, about half the cases in the United States infected with zoonotic *M. leprae* strain*-*types are unable to recall ever having any direct contact with the animals, and other mechanisms also must be involved (Truman et al., 2011).

Investigators have explored the potential role of arthropods in leprosy transmission for more than 80 years. Though conclusive evidence has not been generated, their role has never been fully discounted (Fine, 1982). The potential for arthropod-derived cells to support viable bacteria *in vitro* is supported by the identification of an organism belonging to the *Mycobacterium chelonae* complex being recently isolated while generating a primary cell line from field-caught ticks (Palomar et al., 2019). Likewise, studies have examined the potential for ticks to acquire and transmit *M. leprae* (Souza-Araujo, 1941; Ferreira et al., 2018). In the United States, *Amblyomma* species of ticks transmit a wide variety of infectious organisms and feed on both armadillos and humans (Bechara et al., 2002; Sumner et al., 2007; Harris et al., 2017; Mertins et al., 2017; Suwanbongkot et al., 2019). Specifically, *Amblyomma maculatum* has a distribution which largely overlaps with that of the nine-banded armadillo (Teel et al., 2010; Taulman and Robbins, 2014). The tick’s geographical range is expanding, and includes Florida, Louisiana and Texas, three of the top five states reporting new cases of leprosy (Taulman and Robbins, 2014; HHS, 2015; Sonenshine, 2018). Knowing that *A. maculatum* is an important tick vector of intracellular bacterial pathogens transmissible to vertebrate hosts (Paddock et al., 2004; Ferrari et al., 2012; Grasperge et al., 2014; Banajee et al., 2015; Paddock and Goddard, 2015), this tick as a potential vector for *M. leprae* transmission was examined. In the absence of an available *A. maculatum*-derived cell line, the ISE6, *Ixodes scapularis*-derived cell line was chosen in this study based on the functional use of the cell line for cultivation of other obligate intracellular bacteria (Kurtti et al., 1996; Pornwiroon et al., 2006; Sunyakumthorn et al., 2008). Thus, in the current study we investigated the potential for *A. maculatum* ticks to harbor *M. leprae* and assessed the ISE6 cell line for its potential to propagate bacilli.

## Materials and Methods

### Ticks

A colony of *Mycobacterium*-free *Amblyomma maculatum* ticks was maintained at the Louisiana State University School of Veterinary Medicine as described previously (Grasperge et al., 2014). Briefly, immature and adult tick groups were fed separately via encapsulation on BALB/c mice and Sprague-Dawley rats, respectively. Ticks off host were kept in a 27°C incubator at 90% relative humidity.

### Bacteria

*Mycobacterium leprae* strains; Thai-53 and Br4923 were used in these studies. Both *M. leprae* strains were maintained and isolated from nude mice footpads by the National Hansen’s Disease Program, Laboratory Research Branch (Truman et al., 2004; Montoya et al., 2019).

### Tick Infection Bioassays, Sample Collection, and *Mycobacterium leprae* Detection

Microinjection was employed to expose nymphal and adult ticks as previous described (Kariu et al., 2011), with minor modification for *M. leprae*. Briefly, Drummond 0.4 mm diameter 75 mm length glass capillaries were pulled and fitted with a 0.50 ml Hammond syringe to aspirate a 1:1 dilution consisting of 0.1% Rhodamine B and 1 × 10^9^ bacteria/ml in the *M. leprae* suspension. For ticks injected as nymphs (IAN), a capillary needle was inserted into the anal pore and the suspension (*M. leprae* solution was approximately 5 × 10^3^ *M. leprae*/nymph) injected until the nymph was slightly distended. Immediate mortalities were discarded as they occurred. Injected nymphs were stored in an incubator for 24 h and then assessed for viability and fluorescent detection of Rhodamine B, as described (Harris et al., 2017). Surviving ticks that were positive by fluorescence were allowed to feed to repletion and molt into adults. A portion of these IAN adults were immediately used whole for DNA extraction. Another subsample of IAN adult female ticks was fed with unexposed male ticks for 1, 3, or 6 days before whole ticks were processed for DNA extraction. Individual IAN adult females were allowed to feed until repletion, oviposit, and three pools of either F_1_ larvae (*n* = 30 larvae/pool) or F_1_ nymphs (*n* = 5 nymphs/pool) from individual ticks were placed in TRIzol until DNA extraction, while the remaining F_1_ progeny were allowed to molt to the adult stage. F_1_ adult female ticks were fed with unexposed male ticks for 1, 3, or 6 days before whole ticks were processed for DNA extraction.

Adult tick injections were carried out as described for the nymphs, except ticks were not “filled” until distended (*M. leprae* solution was approximately 5–10 × 10^3^ *M. leprae*/adult tick) and injections were direct into the hemocoel through the caudal margin of the tick, rather than through the anal pore. The injected as adult (IAA) ticks were fed on vertebrate hosts and removed shortly before full engorgement or following natural detachment for saliva collection and tissue recovery. Briefly, ticks were removed from the host and sequentially washed in 70% ethanol and deionized water. Salivation was induced through dorsal application of a 3% pilocarpine in EtOH and collected saliva was pooled into microcentrifuge tubes for subsequent DNA extraction. Subsequently, tick tissues (salivary gland and midgut) were recovered, snap frozen in liquid nitrogen and crushed with a sterile plastic pestle before the addition of TRIzol Reagent (ThermoScientific, CA, United States). and storage at −80°C until DNA extraction previously described (Patton et al., 2012; Suwanbongkot et al., 2019). Samples of vertebrate host skin were collected from the tick feeding site, as well as skin outside the feeding capsule. All skin samples were immediately processed for DNA extraction (Patton et al., 2012; Suwanbongkot et al., 2019). DNA extracted from tick and vertebrate host samples was evaluated for the presence of *M. lepra*e. *Mycobacterium leprae*-specific repetitive element (RLEP) qPCR was used to quantify *M. leprae* as previously described (Truman et al., 2008). Primer sequences were listed in Table 1.

**TABLE 1:** List of primers, sequences, and references used in this study.

Primers	Sequence (5′-3′)	Gene amplified	References
RLEP-F RLEP-R RLEP TaqMan probe	GCAGCAGTATCGTGTTAGTGA CGCTAGAAGGTTGCCGTAT TCGATGATCCGGCCGTCGGCG	*M. leprae*-specific repetitive element (RLEP)	Truman et al., 2008
*esxA*-F *esxA*-R *esxA* probe	CCGAGGGAATAAACCATGCA CGTTTCAGCCGAGTGATTGA 6Fam-TGCTTGCACCAGGTCGCCCA	ESAT-6 protein (*esxA*)	Davis et al., 2013

### Tick Cell Culture and *Mycobacterium leprae* Infection

The ISE6 tick-derived cell line (*Ixodes scapularis*-derived), provided by T. Kurtti (University of Minnesota), was maintained with Leibovitz’s L-15B medium (Gibco-BRL, NY, United States) supplemented with 10% tryptose phosphate broth (pH 6.8–7.0, Sigma, MD, United States), 10% fetal bovine serum (HyClone, UT, United States), and maintained at 32°C in a 5% CO_2_ humidified incubator. For experiments, ISE6 cells at cell density 7 × 10^6^ cells/flask were infected with 1 × 10^8^ *M. leprae/*T25 flask (MOI of 15) and maintained in a 30°C incubator. The presence of *M. leprae* in ISE6 cells was routinely assessed by comparing infected and uninfected cells. Briefly, cells cytocentrifuged onto a slide and stained with Fite’s acid fast staining for 20 min at room temperature (Job and Chacko, 1986). Slides were rinsed with water and 1% acid alcohol was used to decolorize the slides for 1 min or until they were a faint pink. Slides were rinsed again with water, counterstained with methylene blue for 2 min prior to a final rinse, and then mounted in Permount^TM^ (Fisher Scientific). Images were captured using Olympus BX53 microscope.

### Enumeration and Viability Assays for *Mycobacterium leprae* in Tick Cell Culture

ISE6 cells were seeded in 5 ml in a T25 flask at a density of 7 × 10^6^ cells/flask and incubated for 24 h prior to *M. leprae* (Thai-53; 1 × 10^8^) infection. Cells and supernatants were collected at 21, 35, and 49 days post infection (dpi). Briefly, cell pellets and supernatant were collected at each time point, homogenized in a TissueLyser II (QIAGEN, Germany) using a speed setting of 6.5 m/s for 45 s, and nucleic acid was extracted using TRIzol Reagent. Extracted DNA and RNA were stored at −80°C until qPCR was performed. The RLEP qPCR assay previously described for *M. leprae* was used for enumeration (Truman et al., 2008). Normalized *esxA* gene expression was used to determine *M. leprae* viability. Briefly, extracted RNA following DNAse (Fisher Thermo Scientific) treatment and normalization using RLEP counts, was converted to cDNA (cDNA Synthesis Kit-Advantage^®^ RT for PCR kit (Clonetech, CA, United States) and *esxA* gene expression determined by qPCR as previously described (Davis et al., 2013) and sequences of primers are listed in Table 1. Viability in this assay is verified using a standard curve, mock controls in which no reverse transcriptase was used, and positive mouse foot pad controls infected with live *M. leprae*. Two biological replicates for infection assays were performed.

### Transmission Electron Microscopy Imaging

For TEM analysis, ISE6 cells were seeded on plastic cover slips (Thermanox^®^; TMX Coverslips) placed into a 24-well plate at a density of 5 × 10^5^ cells/well. After 24 h, ISE6 cells were infected with freshly isolated *M. leprae* suspension (5 × 10^7^ *M. leprae*/well). Media was removed at 21, 35, and 49 dpi and cover slips were washed with PBS. Fixative solution (3% Glutaraldehyde in 0.1 M CAC buffer, pH 7.4) was added to each well for 30 min at room temperature. Cover slips were then washed three times with 0.1 M CAC buffer with sucrose, pH 7.4 and post fixed with 1% Osmium tetroxide in 0.1 M CAC buffer at room temperature for 1 h. Araldite-Epon 812 (EP) was prepared with 60 g of Aradite (60 g of Polybed 812, 123 g DDSA). Samples were dehydrated with graded series of EtOH (30, 50, 70, 80, 90, and 100%) and embedded gradually with different ratios of EP to EtOH (1:3, 2:2, 3:1, EP only and EP + DMP-30). Sections were then stained with 4% uranyl acetate for 2 min and lead citrate for 1 min. Coverslips and all chemicals were purchased from Electron Microscopy Sciences (Hatfield, PA, United States). Images were captured using TEM: JEOL JEM-1011 microscope (JEOL, Inc., MA).

### Evaluation of *Mycobacterium leprae* Replication and Viability in ISE6 Cells During Extended Culture

*M. leprae* Thai-53 and Br4923 were maintained in ISE6 cells in 30°C incubator and subcultured (1:2) every 8 weeks into uninfected tick cells to avoid overgrowth of ISE6 cells. At approximately 6 month-intervals, *M. leprae* were semi-purified via needle lysis of host cells with low- and high-speed centrifugation (Simser et al., 2001; Sunyakumthorn et al., 2008) and inoculated into T75 uninfected tick cells at cell density of 1.0–1.8 × 10^8^ cells/flask. The cell pellets were collected at 716, 729 dpi for Thai-53 and Br4923 strains, respectively. Samples were processed for imaging as previously detailed.

### Mouse Footpad Infection With *Mycobacterium leprae* Maintained in ISE6 Cells

Both *M. leprae* strains Thai-53 and Br4923 maintained in ISE6 cells for 294 and 309 days, respectively, were purified and inoculated in athymic (nu/nu) mice (Envigo, CA) footpads, to assess their *in vivo* infectivity and growth as previously described (Truman and Krahenbuhl, 2001). Briefly, both hind footpads were inoculated with media alone (control) or either strain of *M. leprae*. The footpads were harvested at 6 months post-inoculation and immediately processed for DNA and RNA isolation to determine *M. leprae* numbers (RLEP) and viability (normalized *esxA* expression).

### Statistical Analysis

Numerical data were analyzed using non-parametric Mann-Whitney test. All statistical analyses were performed with GraphPad Prism 9.0 software.

## Results

### Tick Infection With *Mycobacterium leprae* and Subsequent Transmission

Using the RLEP qPCR, the presence or absence of *M. leprae* in tick samples was assessed after microinjection, during the tick life cycle, and in the vertebrate host skin where the ticks had attached. Of the 29 IAN ticks that molted to the adult stage assessed for *M. leprae* DNA, 71% of the unfed adults were positive (Table 2). Of the ticks that were allowed to feed as adults, 40 and 33% of those that fed for 1 and 6 days, respectively, were positive for *M. leprae* DNA. None of the ticks assessed at 3 days of feeding (*n* = 4) were determined to be infected with *M. leprae*. Additionally, *M. leprae* DNA was detected in the F_1_ larvae and F_1_ nymph pools, but not in the F_1_ adult ticks. For IAN ticks feeding on mice, 50% (*n* = 18) of the skin samples at the tick feeding site were positive for *M. leprae*. A mean (±SD) of 390 (±804) and 630 (±1040) *M. leprae* were detected inside and outside the capsule, respectively. The data suggest that while *M. leprae* can be maintained through transstadial (from nymph to adult) and transovarial (adult female to progeny) transmission events, sustained infection through the entire F_1_ life cycle was not observed.

**TABLE 2:** Tick samples assessed for *M. leprae* infection upon exposure by needle inoculation.

	IAN adults				F_1_ larvae (*n* = 30)	F_1_ nymphs (*n* = 5)	F_1_ adults		
	unfed	day 1	day 3	day6			day 1	day 3	day 6
Total	17	5	4	3	3	3	3	2	2
RLEP positive	12	2	0	1	3	3	0	0	0

*Vertical transmission, from one life cycle stage to the next, was observed for ticks infected as nymphs (IAN), with persistent infection waning by the adult stage of the subsequent generation. The numbers in parentheses are the number of larvae or nymphs per pool.*

The microinjected adult ticks (IAA; *n* = 6) had detectable levels of *M. leprae* in the midgut tissue (∼66%) and salivary gland samples (∼33%), with one tick positive in both tissues. Pooled saliva collected from two adult ticks exposed to *M. leprae* was also positive by PCR.

### *Mycobacterium leprae* Can Infect and Persist in Tick-Derived Cells

**FIGURE 1:**
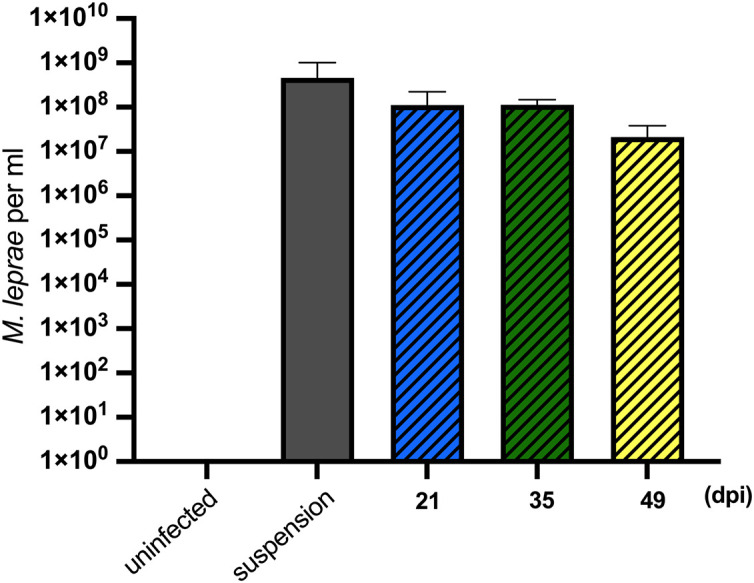
*Mycobacterium leprae* Thai-53 strain enumeration in tick-derived cell lines. ISE6 cells were infected with 1 × 10^8^ *M. leprae*/flask. *Mycobacterium leprae* in cells were harvested at 21, 35, and 49 dpi. *Mycobacterium leprae* DNA quantification was accessed by the RLEP qPCR. Two biological replicates were performed. Statistically significant differences (*p* < 0.05) were analyzed by Mann-Whitney test when comparing with initial suspension.

**FIGURE 2:**
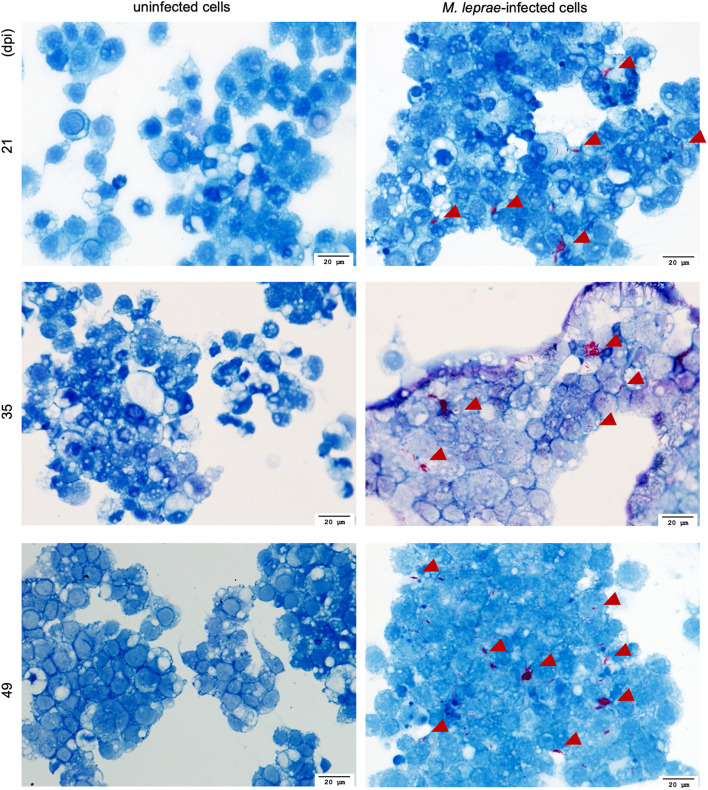
Fite’s Acid fast staining of *M. leprae* Thai-53 strain-infected cells visualization by light microscopy. ISE6 cells were infected with *M. leprae* Thai-53 strain and observed under light microscope at magnification 40× after being stained with Fite’s acid-fast staining at 21, 35, and 49 dpi. Bacilli are shown in red and indicated by arrowheads.

**FIGURE 3:**
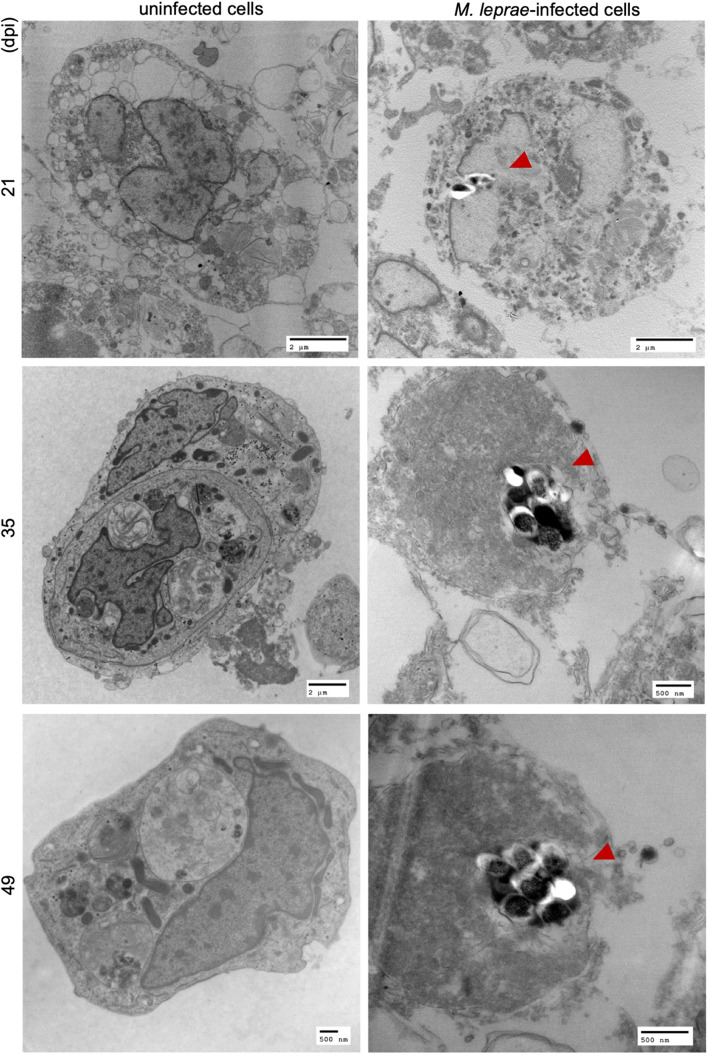
TEM imaging on *M. leprae* Thai-53 strain infected in tick-derived cell lines. ISE6 cells on coverslips were infected with *M. leprae* Thai-53 strain. Infected and uninfected samples at 21, 35, and 49 dpi were prepared for TEM imaging. Images were captured using TEM microscope and bacilli were indicated by red arrowheads.

For successful transmission of *M. leprae*, an obligate intracellular pathogen, the vector must be able to internalize and then, either support growth or maintain viability of the intracellular organisms for a period sufficient to find and transmit to the host. Tick-derived ISE6 cell lines were infected with *M. leprae* (Thai-53) for 21, 35, and 49 days. No significant changes in the *M. leprae* numbers, as enumerated by *M. leprae* specific RLEP qPCR, were observed in tick cell culture at the time points assessed (Figure 1). Both acid fast staining (Figure 2) and TEM (Figure 3) confirmed presence of intracellular bacilli at all these time points. There were no apparent differences in cell morphology or growth characteristics between infected and uninfected ISE6 cells at any of these time points.

### *Mycobacterium leprae* Can Survive in Tick-Derived Cells

**FIGURE 4:**
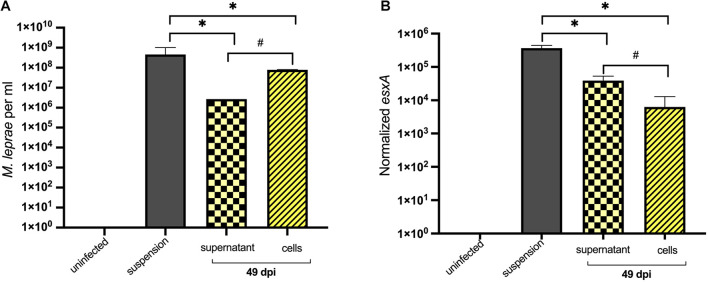
Enumeration and viability of intracellular and extracellular *M. leprae* Thai-53 strain in tick cell culture. ISE6 cells were infected with *M. leprae* at approximately a multiplicity of infection (MOI) of 15. Infected cells and supernatant were collected separately at 49 dpi. **(A)** Enumeration was accessed by qPCR of RLEP. **(B)** *Mycobacterium leprae* viability was determined by detecting *esxA*. Statistically significant differences (Mann-Whitney test; *p* < 0.05) were indicated by ^∗^ or # when comparing with initial suspension or between groups, respectively.

As ISE6 cells were able to maintain *M. leprae* numbers, bacterial viability was assessed by determining the normalized expression of *esxA* gene. Cells and supernatant were collected separately to quantify intracellular and extracellular *M. leprae*, respectively. At 49 days post-inoculation, >90% of the bacteria were intracellular (Figure 4A). Both infected-ISE6 cells and culture supernatant had viable *M. leprae* (Figure 4B). Together these data show that tick derived ISE6 cells do not support propagation but can maintain intracellular *M. leprae* number and viability for at least 49 days.

### Prolonged Maintenance of Viable *Mycobacterium leprae* in Tick-Derived Cells

**FIGURE 5:**
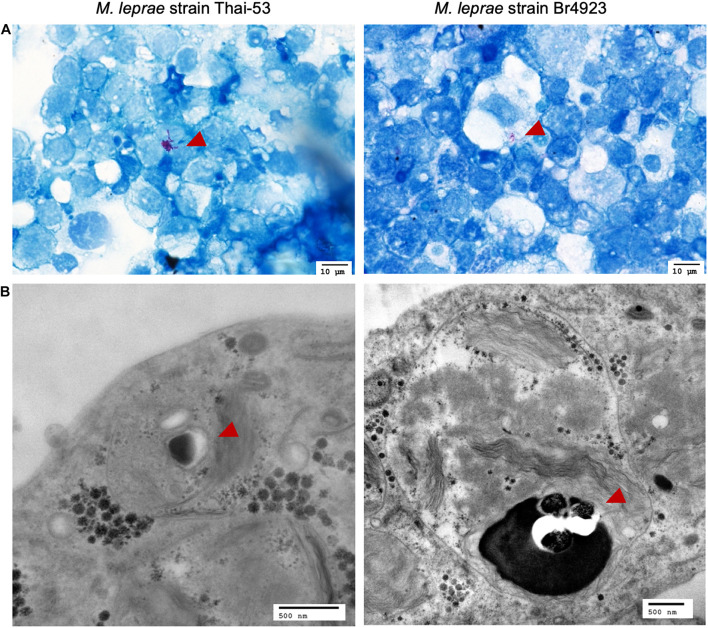
Visualization of *M. leprae*-infected tick cells by light microscopy and TEM. ISE6 cells infected with *M. leprae* Thai-53 and Br4923 strains for over 700 days were visualized but light microscopy at magnification 60× after **(A)** Fite’s acid fast staining and **(B)** by TEM. Bacilli are indicated by red arrowheads.

In addition to the limited time-course infection assay for *M. leprae* in tick cells, bacteria were maintained in cells by regular 1:2 splitting and providing new cells every 8 weeks. Both *M. leprae* strains, Thai-53 and Br4923, were maintained in the laboratory culture for 716 and 729 days, respectively. *Mycobacterium leprae* were observed under light microscopy by acid-fast staining (Figure 5A) and by TEM (Figure 5B), confirming the presence of bacilli in the tick cells after 700 days.

### Tick Cell Cultured *Mycobacterium leprae* Is Infectious for a Vertebrate Host

**FIGURE 6:**
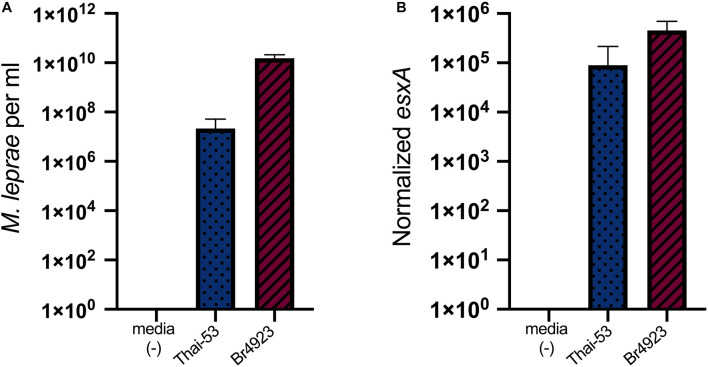
Infectious nature of *M. leprae* after prolonged culture in tick cells. *Mycobacterium leprae*-infected cells maintained for over 300 days were purified and cell-free-*M. leprae* Thai-53 or Br4923 were injected into mouse. Uninfected media served as a negative infection control. After 6 months, *M. leprae* in footpad samples were analyzed by qPCR for bacterial load and viability. **(A)** Enumeration was accessed by qPCR of RLEP. **(B)** *Mycobacterium leprae* viability was determined by detecting *esxA*. Statistically significant differences (*p* < 0.05) were analyzed by Mann-Whitney test when comparing between two strains.

Both *M. leprae* strains Thai-53 and Br4923 were maintained in tick-derived cells for a prolonged period (around 300 days) and then inoculated in athymic nu/nu foot pads. MFPs were harvested 6-month post-inoculation to determine *M. leprae* growth and viability. With no significant differences between strains, both Thai-53 and Br4923 showed appreciable growth (Figure 6A) and were highly viable (Figure 6B) in MFPs. This confirms that tick-derived ISE6 cells were able to maintain intracellular *M. leprae* viability and subsequent infectivity to vertebrate host even after 300 days of *in vitro* culture.

## Discussion

Ticks are effective vectors for transmitting a variety of disease-causing agents, including numerous obligate intracellular bacteria (Laukaitis and Macaluso, 2021). These data indicate that *Amblyomma* ticks infected with *M. leprae* can successfully transmit the organisms both vertically and horizontally, making it possible for them to transfer the organisms to other hosts in subsequent blood meals. Once infected, ticks retain these bacilli through subsequent life-cycle stages and they can pass the organisms along to their progeny. *Mycobacterium leprae* also may have a special affinity for tick cells, as evidenced by the extraordinarily long survival time (≥ 300 days) of the bacilli with *in vitro* cultures of tick cells. In combination, ticks can be effective vehicles aiding the spread of leprosy bacilli between human and zoonotic hosts and their role in perpetuating leprosy merits additional investigation.

Investigating the role ticks may have in the transmission of leprosy is not without precedent. In the 1940s, acid-fast bacilli were found within the gut tissue of *Amblyomma* spp. ticks fed on a leprosy patients’ skin lesion, suggesting the ability to acquire bacilli (Souza-Araujo, 1941). More recently, *Amblyomma sculptum* ticks were determined capable of sustaining *M. leprae* infection (Ferreira et al., 2018). In the United States, *Amblyomma* spp. ticks were recovered from 50% of armadillos trapped, with an average 6 ticks per host (Mertins et al., 2017). Importantly, *A. maculatum*, an aggressive human biting tick has expanding geographical range that overlaps with that of the nine-banded armadillo (Taulman and Robbins, 2014; Sonenshine, 2018).

Employing a microinjection technique to expose ticks to *M. leprae*, nymphs were able to maintain infection and transstadially transmit to the adult stage. Likewise, adult ticks could transovarially transmit *M. leprae* to larvae, which remained infected into the nymphal stage. It has been demonstrated that bacterial loads in ticks can be coupled with the feeding activity (Suwanbongkot et al., 2019); however, feeding F_1_ adult ticks that were infected as nymphs did not result in detectable levels of *M. leprae*. While it is possible that bacilli were shed in the feces of ticks during the feeding process, the presence and viability of *M. leprae* in tick feces requires further examination. Similar infection kinetics were observed for spotted fever group *Rickettsia* in *A. maculatum* ticks (Harris et al., 2017), it is possible that vertebrate amplification hosts may be needed for sustained pathogen infection in tick populations.

Delivery of *M. leprae* via an infected tick may help identify a route of horizontal transmission between zoonotic and human hosts. Of the ticks exposed to *M. leprae* as adults and allowed to take a blood meal, RLEP was detected in midguts and salivary glands after feeding, suggesting organs essential for transmission can become infected. Additionally, pooled saliva collected from these ticks were also positive for RLEP, confirming the potential for horizontal transmission. The detection of *M. leprae* in the skin of vertebrate hosts fed on by recently exposed nymphal ticks further supports the likelihood of horizontal transmission. The results of the current study are consistent with previous examination of *A. sculptum* transmission of *M. leprae* to rabbit hosts (Ferreira et al., 2018). Combined, employing different tick life cycle stages (nymph vs. adult) and inoculation routes (injection vs. feeding), these results demonstrate that ticks can deliver *M. leprae* via saliva during blood meal acquisition. Further studies are needed to determine if ticks can acquire or transmit *M. leprae* while feeding on zoonotic hosts.

A major obstacle to laboratory research on *M. leprae* is associated the difficulty to propagate the organisms in cell culture and the slow doubling time (∼14 days). Utilization of protozoan- and arthropod-derived cell lines have provided an intriguing observation for *M. leprae* cultivation that merits additional consideration (Ferreira et al., 2018). In the current study, *M. leprae* Thai-53 strain was sustained in either tick-derived ISE6 cells at 30°C for up to 49 days. Previously, the ability of tick-derived cells to support viable *M. leprae* Thai-53 identified a cell origin-dependent susceptibility to infection for a 20-day period, with an increase in viable *M. leprae* in *Ixodes*-derived (IDE8) cell line (Ferreira et al., 2018). Despite using *Ixodes*-derived cell lines in both the Ferreira et al. (2018) and the current study, several variables including cell line origin, times of assessment, inoculum dose, and viability RT-qPCR assays likely account for the differences between the two studies. The 16s rRNA/16s rDNA assay employed in the previous study does not account for a minimal threshold of bacteria to assess viability and the residual stability of 16srRNA (Martinez et al., 2009). The viability assay used in the current study requires at least 3,000 bacteria to be present in the reverse transcription reaction in order to evaluate viability (Davis et al., 2013). This assay has been validated by direct comparison with other established viability assays for *M. leprae*, including radiorespirometry, live/dead staining, and mouse foot pad assay. More studies are required to discern if other variables, including host cell background, contribute to the growth kinetics of *M. leprae in vitro*.

Comparison of intracellular and extracellular preparations identified that while *M. leprae* DNA was mostly associated with host cells, viability of *M. leprae* was greater in extracellular supernatant. Because half of the media was replaced on weekly intervals, it is possible that some bacilli were removed from the samples over the course of culture. Subsequent studies should recover any bacilli that may be extracellular and return to the culture during media exchanges to better quantify extracellular *M. leprae*. Bioinformatic analysis indicates that all anabolic pathways of *M. leprae* are intact but non-functional in synthesizing their own energy (Singh and Cole, 2011); therefore, the growth of mycobacteria are restricted by carbon sources and catabolism (Cole et al., 2001; Wheeler, 2001). The L15B media for tick cells is composed of amino acids, tricarboxylic acid, tryptose phosphate, glucose, minerals, vitamins and heat inactivated bovine serum (Munderloh and Kurtti, 1989). The abundant nutrient source can support extracellular survival of other obligate intracellular bacteria including *Rickettsia* (Sunyakumthorn et al., 2008), consistent with what was observed with *M. leprae* for 49 dpi in the current study.

In order to demonstrate *M. leprae* viability, MFP test as gold standard is currently an essential tool in mycobacterium research (Ng et al., 1973; Levy and Ji, 2006). In the current study, after >300 days in culture, both strains of *M. leprae* were able to replicate in MFP, suggesting that non-propagative culture still allows for maintenance of viable bacilli. Ticks can maintain obligate intracellular bacterial populations for an extended life cycle, sometimes up to two years, and transovarially transmit bacteria to their progeny (Laukaitis and Macaluso, 2021). The isolation and cultivation of the *M. chelonae* complex bacilli in tick cells derived from eggs of field-caught ticks (Palomar et al., 2019) supports the current *in vitro* studies demonstrating *Mycobacterium* persistence in tick cells which is consistent with the previous study (Ferreira et al., 2018), *M. leprae* does not show sustainable growth in ISE6 cells. The extended culture of *M. leprae* in tick cells (>700 days) provided an opportunity for visual and molecular characterization. Rare bacilli within tick cells were observed by both acid-fast staining and TEM and observations on *M. leprae* morphology suggest that viable stained bacilli form a rod shape, with dying or dead bacilli commonly appearing as a shorter beaded or granular shape (Scollard et al., 2006). Another observation in prolonged passage of *M. leprae*-infected tick cell culture included the presence of black spheres resembling virus when assessed by TEM. Embryo-derived tick cell lines commonly contain endogenous viruses (Bell-Sakyi and Attoui, 2013); however, the characterization and potential influence of co-infections on *M. leprae* viability or maintenance remain to be examined.

Strain and/or species variation in other tick-borne obligate intracellular bacteria is associated with differences in phenotype in tick cell culture (Bell-Sakyi et al., 2018). After extended culture of *M. leprae* strains Thai-53 and Br4923 in tick-derived cells for ∼300 days, strain Br4923 grew to higher levels in mouse footpads than Thai-53. This would suggest that Br4923 had a higher percent survival rate in the tick cells. Interestingly, when compared head-to-head in an armadillo model, Br4923 likewise exhibited more prolific growth than Thai-53 (Sharma et al., 2018). Although whole genome sequence analysis of these geographically distant *M. leprae* strains suggest they are identical with over 99.995% genome sequence identity in terms of order of genes, sequences, and with no mutations; inversion, translocation, duplication, or transpositions (Monot et al., 2009; Singh and Cole, 2011), maintenance of *M. leprae* in ISE6 cells may select for pathological variations between *M. leprae* strain types. Alternatively, latency, as seen in other *Mycobacterium* spp. including *Mycobacterium tuberculosis*, is a non-metabolically active stage (Cardona and Ruiz-Manzano, 2004) and may have been induced in a strain-specific manner after prolonged culture. However, the ability of *M. leprae* to limit replication in extreme conditions has been shown as bacilli survive in amoebal cysts for months and recover to a replicative state when returned to favorable conditions (Wheat et al., 2014). Nevertheless, the Thai-53 strain recovered from ISE6 cells multiplied once inoculated into the mouse footpads and, like Br4923, produced a robust normalized *esxA* signal indicative of highly viable *M. leprae*.

In summary, the results of the current study demonstrate ticks can harbor *M. leprae* when introduced via needle inoculation. Likewise, the ability to maintain viable and infectious *M. leprae* under culture conditions using an arthropod-derived cell line was exhibited. Identifying the correct pairing of tick cell line and bacterial strain that will facilitate propagation of bacteria will advance the field with respect to molecular biology and therapeutic advances. Similarly, the prolonged culture of *M. leprae* for nearly one year can serve as a model to investigate the biology of pathogen persistence. Although untested in the current study, the evidence for ticks that parasitize both zoonotic reservoir hosts and humans to act as a vector for *M. leprae* is apparent and should be further examined using a biologically relevant transmission/disease system.

## Data Availability Statement

The raw data supporting the conclusions of this article will be made available by the authors, without undue reservation.

## Ethics Statement

The animal study was reviewed and approved by all animal procedures were performed in accordance with the United States Public Health Service Policy on the Humane Care and Use of Laboratory Animals. The use of vertebrate animals for tick feeding was reviewed and approved by the Institutional Animal Care and Use Committee (IACUC) at LSU-SVM (protocol 17-001). The use of mice for the infectivity assay was reviewed and approved by NHDP IACUC (Assurance # D16-00019 [A3032-01]).

## Author Contributions

NT conceived and performed all of the *in vitro* work, qPCR and imaging, with JC and GM. LS and PI performed all of the work with tick vector with the help from JC. MP, RS, RL, LA, and RT provided advice, resources, and support. KM conceived the study and provided advice. NT, LS, and KM worked with RT, RL, and LA to wrote the manuscript, prepared the figures, and tables. All authors provided critical reviews and approved the final draft.

## Conflict of Interest

RS participated in this study while working in the National Hansen’s Disease Program and is currently employed by Retrobiotech LLC., United States. The remaining authors declare that the research was conducted in the absence of any commercial or financial relationships that could be construed as a potential conflict of interest.

## Publisher’s Note

All claims expressed in this article are solely those of the authors and do not necessarily represent those of their affiliated organizations, or those of the publisher, the editors and the reviewers. Any product that may be evaluated in this article, or claim that may be made by its manufacturer, is not guaranteed or endorsed by the publisher.

## References

[B1] Avanzi C. Del-Pozo J. Benjak A. Stevenson K. Simpson V. R. Busso P. (2016). Red squirrels in the British Isles are infected with leprosy bacilli. *Science* 354 744–747. 10.1126/science.aah3783 27846605

[B2] Banajee K. H. Embers M. E. Langohr I. M. Doyle L. A. Hasenkampf N. R. Macaluso K. R. (2015). Correction: amblyomma maculatum feeding augments rickettsia parkeri infection in a rhesus macaque model: a pilot study. *PLoS One* 10:e0137598. 10.1371/journal.pone.0137598 26244337 PMC4526656

[B3] Bechara G. H. Szabo M. P. Almeida Filho W. V. Bechara J. N. Pereira R. J. Garcia J. E. (2002). Ticks associated with armadillo (Euphractus sexcinctus) and anteater (*Myrmecophaga tridactyla*) of emas national park, state of goias, brazil. *Ann. N Y Acad. Sci.* 969 290–293. 10.1111/j.1749-6632.2002.tb04394.x 12381607

[B4] Bell-Sakyi L. Attoui H. (2013). Endogenous tick viruses and modulation of tick-borne pathogen growth. *Front. Cell Infect. Microbiol.* 3:25. 10.3389/fcimb.2013.00025 23875176 PMC3709243

[B5] Bell-Sakyi L. Darby A. Baylis M. Makepeace B. L. (2018). The Tick Cell Biobank: A global resource for in vitro research on ticks, other arthropods and the pathogens they transmit. *Ticks Tick Borne Dis.* 9 1364–1371. 10.1016/j.ttbdis.2018.05.015 29886187 PMC6052676

[B6] Blake L. A. West B. C. Lary C. H. Todd J. R. (1987). Environmental nonhuman sources of leprosy. *Rev. Infect. Dis.* 9 562–577. 10.1093/clinids/9.3.562 3299637

[B7] Cardona P. J. Ruiz-Manzano J. (2004). On the nature of Mycobacterium tuberculosis-latent bacilli. *Eur. Respir. J.* 24 1044–1051. 10.1183/09031936.04.00072604 15572551

[B8] Cole S. T. Eiglmeier K. Parkhill J. James K. D. Thomson N. R. Wheeler P. R. (2001). Massive gene decay in the leprosy bacillus. *Nature* 409 1007–1011. 10.1038/35059006 11234002

[B9] Davey T. F. (1974). The nasal discharge in leprosy. *Nurs. J. India* 65:20. 4596613

[B10] Davis G. L. Ray N. A. Lahiri R. Gillis T. P. Krahenbuhl J. L. Williams D. L. (2013). Molecular assays for determining *Mycobacterium leprae* viability in tissues of experimentally infected mice. *PLoS Negl. Trop. Dis.* 7:e2404. 10.1371/journal.pntd.0002404 24179562 PMC3750008

[B11] Desikan K. V. Sreevatsa (1995). Extended studies on the viability of Mycobacterium leprae outside the human body. *Lepr. Rev.* 66 287–295. 8637382

[B12] Ferrari F. A. Goddard J. Paddock C. D. Varela-Stokes A. S. (2012). Rickettsia parkeri and candidatus rickettsia andeanae in gulf coast ticks. mississippi, USA. *Emerg. Infect. Dis.* 18 1705–1707. 10.3201/eid1810.120250 23018026 PMC3471625

[B13] Ferreira J. D. S. Souza Oliveira D. A. Santos J. P. Ribeiro C. C. D. U. Baêta B. A. Teixeira R. C. (2018). Ticks as potential vectors of Mycobacterium leprae: Use of tick cell lines to culture the bacilli and generate transgenic strains. *PLoS Negl. Trop. Dis.* 12:e0007001. 10.1371/journal.pntd.0007001 30566440 PMC6326517

[B14] Fine P. E. (1982). Leprosy: the epidemiology of a slow bacterium. *Epidemiol. Rev.* 4 161–188. 10.1093/oxfordjournals.epirev.a036245 6754406

[B15] Grasperge B. J. Morgan T. W. Paddock C. D. Peterson K. E. Macaluso K. R. (2014). Feeding by Amblyomma maculatum (Acari: Ixodidae) enhances Rickettsia parkeri (Rickettsiales: Rickettsiaceae) infection in the skin. *J. Med. Entomol.* 51 855–863. 10.1603/me13248 25118419 PMC4214552

[B16] Harris E. K. Verhoeve V. I. Banajee K. H. Macaluso J. A. Azad A. F. Macaluso K. R. (2017). Comparative vertical transmission of rickettsia by dermacentor variabilis and amblyomma maculatum. *Ticks Tick Borne Dis.* 8 598–604. 10.1016/j.ttbdis.2017.04.003 28433729 PMC5702269

[B17] HHS (2015). *A Summary of Hansen’s Disease in the United States-2014.* Washington, DC: U.S. Department of Health and Human Services.

[B18] Job C. K. Chacko C. J. (1986). A modification of Fite’s stain for demonstration of M. leprae in tissue sections. *Indian J. Lepr.* 58 17–18. 2427624

[B19] Kariu T. Coleman A. S. Anderson J. F. Pal U. (2011). Methods for rapid transfer and localization of lyme disease pathogens within the tick gut. *J. Vis. Exp.* 48:2544. 10.3791/2544 21372782 PMC3280631

[B20] Kurtti T. J. Munderloh U. G. Andreadis T. G. Magnarelli L. A. Mather T. N. (1996). Tick cell culture isolation of an intracellular prokaryote from the tick Ixodes scapularis. *J. Invertebr. Pathol.* 67 318–321. 10.1006/jipa.1996.0050 8812616

[B21] Laukaitis H. J. Macaluso K. R. (2021). Unpacking the intricacies of Rickettsia-vector interactions. *Trends Parasitol.* 37 734–746. 10.1016/j.pt.2021.05.008 34162522 PMC8344978

[B22] Levy L. Ji B. (2006). The mouse foot-pad technique for cultivation of Mycobacterium leprae. *Lepr. Rev.* 77 5–24. 16715686

[B23] Martinez A. N. Lahiri R. Pittman T. L. Scollard D. Truman R. Moraes M. O. (2009). Molecular determination of Mycobacterium leprae viability by use of real-time PCR. *J. Clin. Microbiol.* 47 2124–2130. 19439537 10.1128/JCM.00512-09 PMC2708532

[B24] Mertins J. W. Vigil S. L. Corn J. L. (2017). Amblyomma auricularium (Ixodida: Ixodidae) in Florida: new hosts and distribution records. *J. Med. Entomol.* 54 132–141. 10.1093/jme/tjw159 28082640 PMC6457082

[B25] Mohanty P. S. Naaz F. Katara D. Misba L. Kumar D. Dwivedi D. K. (2016). Viability of Mycobacterium leprae in the environment and its role in leprosy dissemination. *Indian J. Dermatol. Venereol. Leprol.* 82 23–27. 10.4103/0378-6323.168935 26728806

[B26] Monot M. Honoré N. Garnier T. Zidane N. Sherafi D. Paniz-Mondolfi A. (2009). Comparative genomic and phylogeographic analysis of Mycobacterium leprae. *Nat. Genet.* 41 1282–1289. 10.1038/ng.477 19881526

[B27] Montoya D. J. Andrade P. Silva B. J. A. Teles R. M. B. Ma F. Bryson B. (2019). Dual RNA-seq of human leprosy lesions identifies bacterial determinants linked to host immune response. *Cell Rep.* 26 3574–3585. 10.1016/j.celrep.2019.02.109 30917313 PMC6508871

[B28] Munderloh U. G. Kurtti T. J. (1989). Formulation of medium for tick cell culture. *Exp. Appl. Acarol.* 7 219–229. 10.1007/BF01194061 2766897

[B29] Ng H. Jacobsen P. L. Levy L. (1973). Analogy of Mycobacterium marinum disease to Mycobacterium leprae infection in footpads of mice. *Infect. Immun.* 8 860–867. 10.1128/iai.8.6.860-867.1973 4594116 PMC422941

[B30] Paddock C. D. Goddard J. (2015). The evolving medical and veterinary importance of the gulf coast tick (Acari: Ixodidae). *J. Med. Entomol.* 52 230–252. 10.1093/jme/tju022 26336308

[B31] Paddock C. D. Sumner J. W. Comer J. A. Zaki S. R. Goldsmith C. S. Goddard J. (2004). Rickettsia parkeri: a newly recognized cause of spotted fever rickettsiosis in the United States. *Clin. Infect. Dis.* 38 805–811. 10.1086/381894 14999622

[B32] Palomar A. M. Premchand-Branker S. Alberdi P. Belova O. A. Moniuszko-Malinowska A. Kahl O. (2019). Isolation of known and potentially pathogenic tick-borne microorganisms from European ixodid ticks using tick cell lines. *Ticks Tick Borne Dis.* 10 628–638. 10.1016/j.ttbdis.2019.02.008 30819609 PMC6446187

[B33] Patton T. G. Dietrich G. Brandt K. Dolan M. C. Piesman J. Gilmore R. D. (2012). Saliva, salivary gland, and hemolymph collection from Ixodes scapularis ticks. *J. Vis. Exp.* 60:3894. 10.3791/3894 22371172 PMC3912584

[B34] Pornwiroon W. Pourciau S. S. Foil L. D. Macaluso K. R. (2006). Rickettsia felis from cat fleas: isolation and culture in a tick-derived cell line. *Appl. Environ. Microbiol.* 72 5589–5595. 10.1128/AEM.00532-06 16885313 PMC1538700

[B35] Predaswat P. B. (1992). *Khi Thut, “The Disease of Social Loathing”: An Anthropology of the Stigma of Leprosy in Rural Northeast Thailand.* San Francisco: University of California.

[B36] Scollard D. M. (2005). Leprosy research declines, but most of the basic questions remain unanswered. *Int. J. Lepr. Mycobact. Dis.* 73 25–27. 10.1489/1544-581X(2005)73[25:LRDBMO]2.0.CO;2 15898836

[B37] Scollard D. M. Adams L. B. Gillis T. P. Krahenbuhl J. L. Truman R. W. Williams D. L. (2006). The continuing challenges of leprosy. *Clin. Microbiol. Rev.* 19 338–381. 16614253 10.1128/CMR.19.2.338-381.2006 PMC1471987

[B38] Sharma R. Singh P. Loughry W. J. Lockhart J. M. Inman W. B. Duthie M. S. (2015). Zoonotic leprosy in the southeastern United States. *Emerg. Infect. Dis.* 21 2127–2134. 10.3201/eid2112.150501 26583204 PMC4672434

[B39] Sharma R. Singh P. Pena M. Subramanian R. Chouljenko V. Kim J. (2018). Differential growth of Mycobacterium leprae strains (SNP genotypes) in armadillos. *Infect. Genet. Evol.* 62 20–26. 10.1016/j.meegid.2018.04.017 29665434

[B40] Simser J. A. Palmer A. T. Munderloh U. G. Kurtti T. J. (2001). Isolation of a spotted fever group Rickettsia, Rickettsia peacockii, in a Rocky Mountain wood tick, Dermacentor andersoni, cell line. *Appl. Environ. Microbiol.* 67 546–552. 10.1128/AEM.67.2.546-552.2001 11157215 PMC92619

[B41] Singh P. Cole S. T. (2011). Mycobacterium leprae: genes, pseudogenes and genetic diversity. *Future Microbiol.* 6 57–71. 10.2217/fmb.10.153 21162636 PMC3076554

[B42] Sonenshine D. E. (2018). Range expansion of tick disease vectors in north america: implications for spread of tick-borne disease. *Int. J. Environ. Res. Public Health* 15:478. 10.3390/ijerph15030478 29522469 PMC5877023

[B43] Souza-Araujo H. C. (1941). Poderá o carrapato transmitir a lepra. *Memorias Do Instituto Oswaldo Cruz* 36 577–584.

[B44] Sumner J. W. Durden L. A. Goddard J. Stromdahl E. Y. Clark K. L. Reeves W. K. (2007). Gulf Coast ticks (Amblyomma maculatum) and Rickettsia parkeri. *US Emerg. Infect. Dis.* 13 751–753. 10.3201/eid1305.061468 17553257 PMC2738460

[B45] Sunyakumthorn P. Bourchookarn A. Pornwiroon W. David C. Barker S. A. Macaluso K. R. (2008). Characterization and growth of polymorphic Rickettsia felis in a tick cell line. *Appl. Environ. Microbiol.* 74 3151–3158. 10.1128/AEM.00025-08 18359823 PMC2394910

[B46] Suwanbongkot C. Langohr I. M. Harris E. K. Dittmar W. Christofferson R. C. Macaluso K. R. (2019). Spotted Fever Group. *Infect. Immun.* 87:18. 10.1128/IAI.00804-18 30642897 PMC6434108

[B47] Taulman J. F. Robbins L. W. (2014). Range expansion and distributional limits of the nine-banded armadillo in the united states: an update of taulman & robbins (1996). *J. Biogeograp.* 41 1626–1630. 10.1111/jbi.12319

[B48] Teel P. D. Ketchum H. R. Mock D. E. Wright R. E. Strey O. F. (2010). The Gulf Coast tick: a review of the life history, ecology, distribution, and emergence as an arthropod of medical and veterinary importance. *J. Med. Entomol.* 47 707–722. 10.1603/me10029 20939363

[B49] Tio-Coma M. Wijnands T. Pierneef L. Schilling A. K. Alam K. Roy J. C. (2019). Detection of Mycobacterium leprae DNA in soil: multiple needles in the haystack. *Sci. Rep.* 9:3165. 10.1038/s41598-019-39746-6 30816338 PMC6395756

[B50] Truman R. Fine P. E. (2010). ‘Environmental’ sources of Mycobacterium leprae: issues and evidence. *Lepr. Rev.* 81 89–95. 20825112

[B51] Truman R. Fontes A. B. De Miranda A. B. Suffys P. Gillis T. (2004). Genotypic variation and stability of four variable-number tandem repeats and their suitability for discriminating strains of Mycobacterium leprae. *J. Clin. Microbiol.* 42 2558–2565. 10.1128/JCM.42.6.2558-2565.2004 15184434 PMC427888

[B52] Truman R. W. Andrews P. K. Robbins N. Y. Adams L. B. Krahenbuhl J. L. Gillis T. P. (2008). Enumeration of *Mycobacterium leprae* using real-time PCR. *PLoS Negl. Trop. Dis.* 2:e328. 10.1371/journal.pntd.0000328 18982056 PMC2570796

[B53] Truman R. W. Krahenbuhl J. L. (2001). Viable *M. leprae* as a research reagent. *Int. J. Lepr. Mycobact. Dis.* 69 1–12. 11480310

[B54] Truman R. W. Shannon E. J. Hagstad H. V. Hugh-Jones M. E. Wolff A. Hastings R. C. (1986). Evaluation of the origin of Mycobacterium leprae infections in the wild armadillo, *Dasypus novemcinctus*. *Am. J. Trop. Med. Hyg.* 35 588–593. 3518509 10.4269/ajtmh.1986.35.588

[B55] Truman R. W. Singh P. Sharma R. Busso P. Rougemont J. Paniz-Mondolfi A. (2011). Probable zoonotic leprosy in the southern United States. *N. Engl. J. Med.* 364 1626–1633. 10.1056/NEJMoa1010536 21524213 PMC3138484

[B56] Walker S. L. Lockwood D. N. (2006). The clinical and immunological features of leprosy. *Br. Med. Bull.* 2006:10. 10.1093/bmb/ldl010 17090777

[B57] Wheat W. H. Casali A. L. Thomas V. Spencer J. S. Lahiri R. Williams D. L. (2014). Long-term survival and virulence of Mycobacterium leprae in amoebal cysts. *PLoS Negl. Trop. Dis.* 8:e3405. 10.1371/journal.pntd.0003405 25521850 PMC4270725

[B58] Wheeler P. R. (2001). The microbial physiologist’s guide to the leprosy genome. *Lepr. Rev.* 72 399–407. 10.5935/0305-7518.20010048 11826476

[B59] World Health Organization [WHO] (2019). *Meeting of the International Task Force for Disease Eradication, April 2018.* Geneva: World Health Organization.

